# A *trans*-activator-like structure in RCNMV RNA1 evokes the origin of the *trans*-activator in RNA2

**DOI:** 10.1371/journal.ppat.1008271

**Published:** 2020-01-06

**Authors:** Laura R. Newburn, K. Andrew White

**Affiliations:** Department of Biology, York University, Toronto, Ontario, Canada; Agriculture and Agri-Food Canada, CANADA

## Abstract

The Red clover necrotic mosaic virus (RCNMV) genome consists of two plus-strand RNA genome segments, RNA1 and RNA2. RNA2 contains a multifunctional RNA structure known as the *trans*-activator (TA) that (i) promotes subgenomic mRNA transcription from RNA1, (ii) facilitates replication of RNA2, and (iii) mediates particle assembly and copackaging of genome segments. The TA has long been considered a unique RNA element in RCNMV. However, by examining results from RCNMV genome analyses in the ViRAD virus (re-)annotation database, a putative functional RNA element in the polymerase-coding region of RNA1 was identified. Structural and functional analyses revealed that the novel RNA element adopts a TA-like structure (TALS) and, similar to the requirement of the TA for RNA2 replication, the TALS is necessary for the replication of RNA1. Both the TA and TALS possess near-identical asymmetrical internal loops that are critical for efficient replication of their corresponding genome segments, and these structural motifs were found to be functionally interchangeable. Moreover, replacement of the TA in RNA2 with a stabilized form of the TALS directed both RNA2 replication and packaging of both genome segments. Based on their comparable properties and considering evolutionary factors, we propose that the TALS appeared *de novo* in RNA1 first and, subsequently, the TA arose *de novo* in RNA2 as a functional mimic of the TALS. This and other related information were used to formulate a plausible evolutionary pathway to describe the genesis of the bi-segmented RCNMV genome. The resulting scenario provides an evolutionary framework to further explore and test possible origins of this segmented RNA plant virus.

## Author summary

We have identified a novel RNA element in RNA1 of the segmented positive-strand RNA genome of Red clover necrotic mosaic virus (RCNMV). Its similarity in structure to a known important RNA element in RNA2 suggests that the two RNA structures perform similar viral functions in their respective genome segments, and this was confirmed experimentally. Based on evolutionary considerations, the RNA element in RNA1 is proposed to have appeared earlier in RCNMV genome genesis, with the corresponding element in RNA2 arising subsequently as a functional mimic of the former. This information was integrated into a plausible pathway for the emergence and evolution of this segmented viral genome, thereby providing novel insights into the puzzling history of RCNMV.

## Introduction

**Fig 1:**
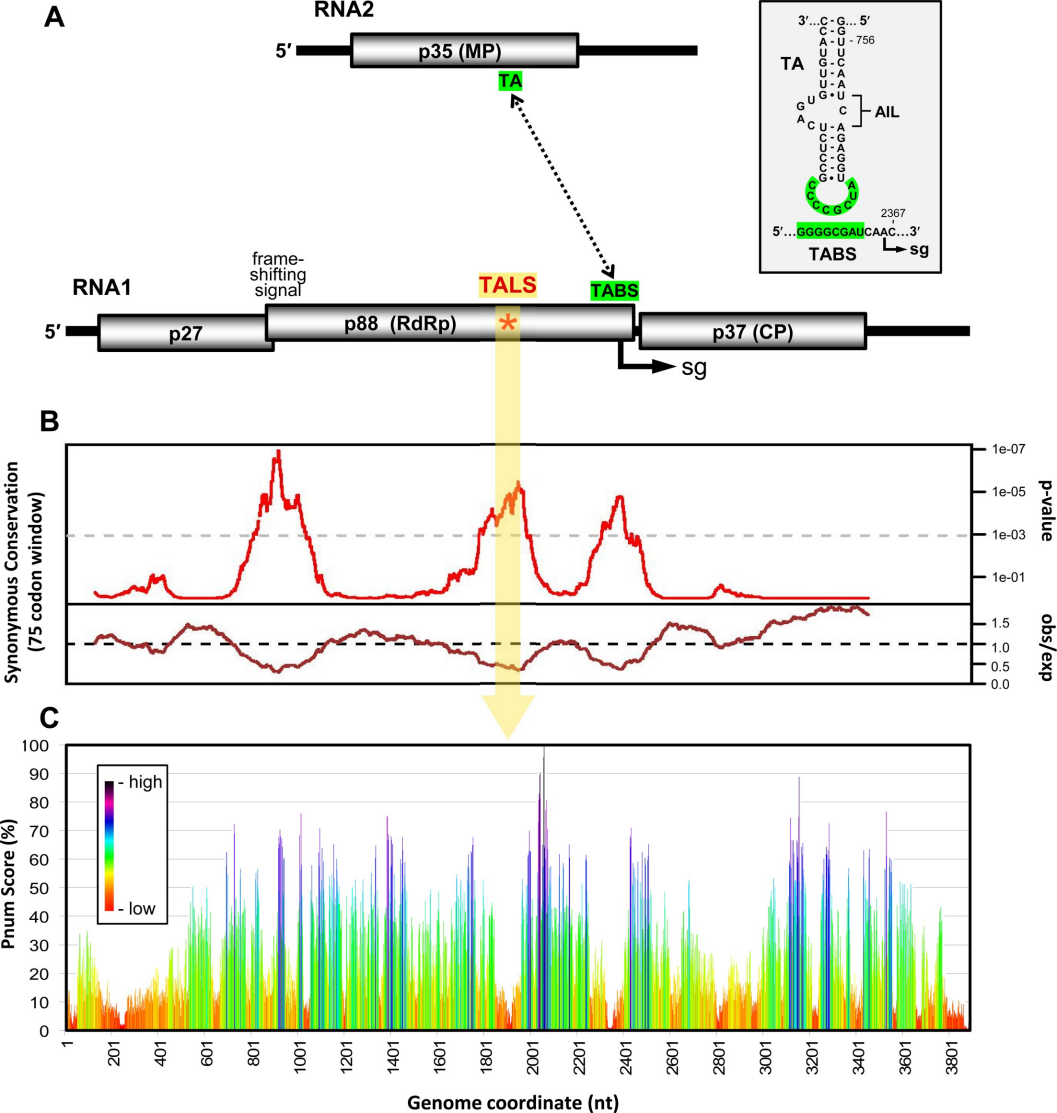
RCNMV genome organization, including synonymous site and structural analyses. **(A)** Schematics of RCNMV RNA1 and RNA2 genome structures. Encoded proteins are shown as grey cylinders with the sizes of corresponding proteins indicated in kilodaltons. The TA in RNA2 and the TABS in RNA1 are indicated, with a dotted line signifying their complementarity. The position of the TALS structure is indicated by a red asterisk, and the transcription initiation site of the sg mRNA in RNA1 is shown as a bent arrow labelled sg. The inset shows the nucleotide sequences of the TA and TABS. The complementary sequences in both RNAs are shaded in green and the AIL in the TA is denoted by a bracket. **(B)** Synonymous site conservation analysis of RCNMV RNA1. Results from a 75-codon sliding window are shown, with observed-over-expected rates of synonymous site conservation in the bottom graph and corresponding p-values in the upper graph. The position corresponding to the TALS in RNA1 in (A) is indicated by a vertical yellow band. The graph was reproduced from the ViRAD virus (re-)annotation database [20]. Analysis was performed using RNA1 sequences from RCNMV-Can (Genbank accession number, AB034916), RCNMV-Aus (J04357) and SCNMV (L07884). See text for details. **(C)** Pnum analysis of RCNMV RNA1 using Mfold [22]. Regions that are predicted to be well defined in the structure correspond to low Pnum values, and are depicted in red and orange. See text for details.

*Dianthovirus* is the only genus in the family *Tombusvididae* that possesses a segmented genome and, as such, its members face unique challenges [1]. There are currently three approved dianthoviruses, the type species Carnation ringspot virus (CRSV), Sweet clover necrotic mosaic virus (SCNMV), and Red clover necrotic mosaic virus (RCNMV) [2–4]. These viruses contain plus-strand RNA genomes that are composed of two segments, which are copackaged into ~36 nm icosahedral particles. The best studied of these, RCNMV, is composed of RNA1 (3.9 kb) and RNA2 (1.45 kb), and neither genome segment is 5ꞌ-capped or 3ꞌ-polyadenylated (**Fig 1A**) [2, 3]. The translation of proteins from RNA1 involves a 3ꞌ cap-independent translational enhancer in its 3ꞌ-untranslated region (UTR) [5, 6], while the translational mechanism utilized by RNA2 is unclear [7]. An accessory replication protein, p27, is encoded at the 5ꞌ-end of RNA1, and the p88 RNA-dependent RNA polymerase (RdRp) is produced as a C-terminal extension of p27 via translational frameshifting [8–12]. The 3ꞌ-third of RNA1 encodes the coat protein (CP), which is translated from a subgenomic (sg) mRNA that is transcribed during infections [13]. RNA2 encodes only p35, which facilitates virus movement within plants [14].

Production of the CP-encoding sg mRNA requires an inter-genomic 8 bp-long interaction between the terminal loop of a stem-loop structure in RNA2, called the *trans*-activator (TA), and a sequence in RNA1, the TA binding site (TABS), located just upstream from the transcription start site [13] (**Fig 1A**). This interaction forms a structure that blocks the RdRp’s progress during minus-strand synthesis of RNA1 [15], which generates a sg mRNA-sized minus-strand RNA that serves as the template for sg mRNA transcription (**S1 Fig**). Additionally, because the *trans*-interaction is concentration dependent, it coordinates CP production with high levels of both genome segments later in infections [13].

The TA-TABS interaction between RNA1 and RNA2 is also proposed to facilitate copackaging of the two segments into the same particle [16], and the TA has been implicated as the packaging signal for virus assembly [17]. The latter function is consistent with *in vitro* assays showing that RNA fragments of the TA alone or a TA-TABS complex bind preferentially to recombinant CP, however the precise regions of CP contact in these RNAs were not defined [18]. In addition to these assembly-related functions, the TA is required for replication of RNA2 [19]. Exactly how this latter role is accomplished is not known, but it likely involves the TA interacting with a viral or host protein(s) via its asymmetrical internal loop (AIL) and/or terminal loop (**Fig 1A, inset**).

In this study we have identified an RNA structure in RNA1 that has both structural and functional similarities to the TA, and, based on its likeness to the TA, the new element was termed the TA-like structure (TALS). Here we describe the discovery and analysis of this new RNA replication element, and compare and contrast its properties with those of the TA. The parallels between these RNA elements suggest an intriguing relationship, which we discuss with respect to the genesis of the RCNMV genome.

## Results

### Identification of a candidate functional RNA structure in RCNMV RNA1

The ViRAD virus (re-)annotation database provides information on the likely presence of overlapping ORFs or functional RNA elements within the coding regions of viral RNA genomes [20]. These data sets were generated by comparing the coding regions of genomes from related viral species and/or strains and identifying regions that exhibit greater than expected rates of conservation at synonymous sites; which is indicative of purifying selection to maintain function (**Fig 1B, lower graph**). Regions with p-values corresponding to 0.001 (1e-03, grey dotted line) or less are considered to be significant and represent prime candidates for harboring functionally relevant RNA elements. Results from this analysis of dianthovirus RNA1s revealed three regions meeting the significance threshold that corresponded to the frameshifting site, an internal segment in the p88 ORF, and the TABS region (**Fig 1B, upper graph**). The first and last regions were expected, as their positions overlapped with previously reported functional RNA structures [12, 13]. However, the middle sequence in the p88 coding region did not correspond to any known RNA elements.

The entire RCNMV RNA1 genome was also computationally-folded and analyzed for the conservation of RNA secondary structures in optimal and suboptimal folds [21]. Well-maintained structures within an ensemble of lowest free energy folds are inversely proportional to a calculated Pnum score. The results for RNA1 indicated that the central region that showed a high level of synonymous site conservation (**Fig 1B**) coincided with a section of the genome that exhibited well-defined secondary structure (*i*.*e*., a low Pnum score, red/orange) (**Fig 1C**). These results are consistent with the existence of a functional RNA structure located somewhere between nucleotides 1800 and 2000 in RNA1.

### Structural characterization of the novel RNA element in RNA1 and its prevalence in dianthoviruses

**Fig 2:**
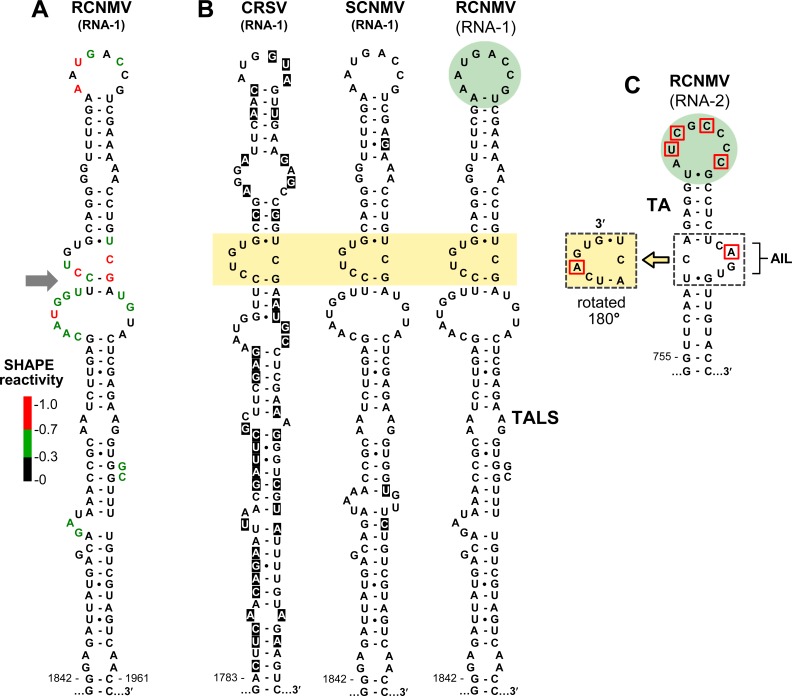
Comparison of the RNA structures of the TALS and TA. **(A)** SHAPE analysis of RCNMV TALS RNA. The relative reactivity of each nucleotide is indicated, where red, green, and black nucleotides represent highly, moderately, and poorly reactive residues, respectively. The grey arrow to the left shows a region in the TALS structure that is predicted to be dynamic. The two base pairs below the AIL in the TALS are shown as paired to facilitate comparison with that in the TA, though this interaction is likely dynamic. **(B)** Comparison of TALS RNA structures in the RNA1s of three dianthoviruses. Nucleotide variability is shown with black highlights and the conserved AILs as well as their closing base pairs are shaded yellow. The terminal loop of the TALS in RCNMV RNA1 is shaded green to indicate its correspondence with the terminal loop of the TA in RNA2. **(C)** The TA in RNA2 of RCNMV. The AIL and closing base pairs are denoted by a white dotted box and their rotation by 180° is shown to the left in a yellow box. The green shading denotes the terminal loop of the TA and the nucleotides boxed in red differ in sequence from the corresponding nucleotides in the TALS.

Computational RNA folding of RCNMV RNA1 predicted an extended stem loop structure spanning coordinates 1841 to 1962, and corresponding solution structure mapping of this region within the context of a full-length RNA1 supported the proposed structure (**Fig 2A**). Selective 2ꞌ-hydroxyl acylation analyzed by primer extension (SHAPE) revealed high levels of reactivity in predicted single-stranded regions and minimal modifications for base paired residues. The varied reactivities observed in the region modeled to form a two-base-pair stem indicates that this interaction may be dynamic (**Fig 2A, grey arrow**).

Similar extended stem loop structures were also predicted in other sequenced species of *Dianthovirus*, namely CRSV and SCNMV (**Fig 2B**). While the general RNA conformation was maintained in all three viruses, most of the helical interruptions (*i*.*e*., mismatches, bulges, and internal loops), were not conserved. One exception to this trend was a centrally-positioned asymmetrical internal loop (AIL) and its closing base pairs that was present in all species (**Fig 2B, yellow shading**). In CRSV, the stem below its AIL was extended by two canonical base pairs, supporting the AIL’s existence and suggesting that pairing also occurs below the AILs in SCNMV and RCNMV.

Interestingly, the RNA1 AILs displayed a close resemblance to the AIL present in the TA in RNA2 of RCNMV (**Fig 2C, white box**), which is more apparent when the latter is rotated by 180° relative to its stem (**Fig 2C, yellow box**). Small differences in this region between the two genome segments included an adenylate in the TA’s AIL in RNA2 that was a uridylate in the RNA1 structures, and alternative closing pyrimidine-purine base pairs at one end of the AILs (*i*.*e*., U-A versus C-G) (**Fig 2B and 2C**). The RCNMV RNA1 structure and TA in RNA2 also possessed a second structural feature that was comparable; an eight-residue terminal loop, in which half of the nucleotides were identical (**Fig 2B and 2C, green circles**). Based on both internal and terminal structural similarities to the TA, the novel RNA1 structure was named TA-like structure or TALS.

### The AIL in the TALS is essential for RNA1 replication in infections

**Fig 3:**
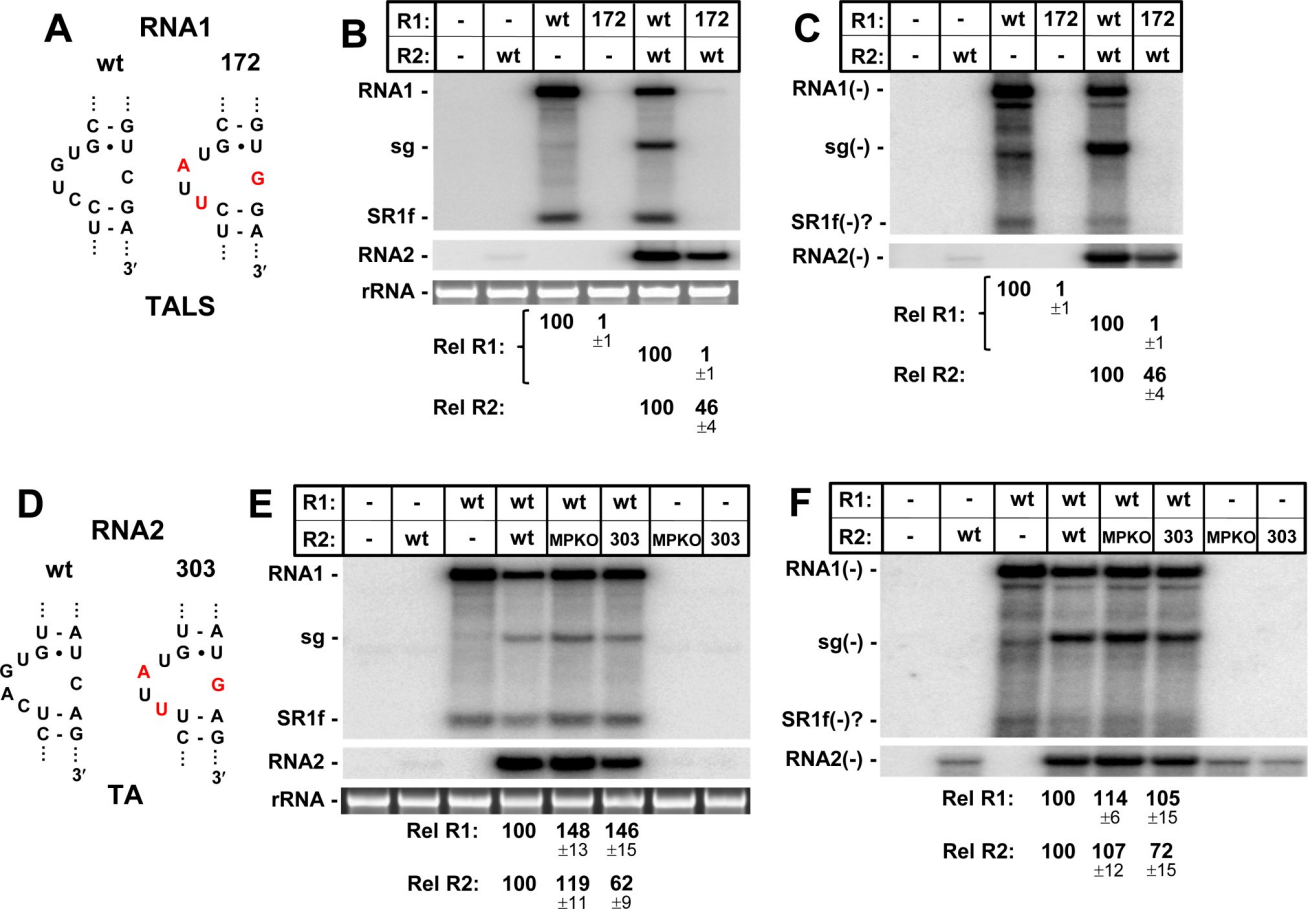
Mutational analysis of the AILs in the TALS and TA in RCNMV. **(A)** Wt and mutated AIL in the TALS of RNA1, with substituted residues in red. **(B)** Corresponding northern blot analysis of RCNMV plus-strand RNA accumulation in plant protoplast transfections incubated for 22 hours at 17°C. Shown above each lane is the name of the viral genome included in the infection. The positions of the viral genome segments (RNA1 and RNA2), the sg mRNA (sg), and a 3ꞌ-degradation product of RNA1 (SR1f) [22] are indicated to the left of the blot. Corresponding loading controls (25S rRNA) are shown for each lane. The relative RNA1 (Rel. RNA1) and RNA2 (Rel. RNA2) levels are provided below, with corresponding means (± standard error) calculated from three independent protoplast infections. **(C)** Corresponding northern blot analysis of RCNMV minus-strand RNA accumulation in plant protoplasts. **(D)** Wt and mutated AIL in the TA of RNA2, with substituted residues in red. **(E)** Corresponding northern blot analysis of RCNMV plus-strand RNA accumulation in plant protoplasts. **(F)** Corresponding northern blot analysis of RCNMV minus-strand RNA accumulation in plant protoplasts. Trace levels of minus-strands detected in RNA2-only lanes is likely due to low level T7-polymerase switching to the non-template strand during in vitro transcription of RNA2.

The conservation of the AIL in the TALS of different species and its similarity to the AIL in the TA prompted further investigation of its possible function. To address this, three silent nucleotide substitutions were introduced into the AIL in RCNMV RNA1, creating mutant R1-172 (**Fig 3A**). Transfection of R1-172 alone into plant protoplasts and subsequent northern blot analyses revealed that the introduced changes eliminated its accumulation (**Fig 3B**). In cotransfections of R1-172 with wt RNA2, RNA2 replicated to notable levels, indicating that the mutations in R1-172 did not inhibit translation of replication proteins p27 and p88, and that, even though it was not amplifiable, the mutant was nonetheless stable (**Fig 3B**). These results indicate that the inhibition observed is related to genome replication, and analysis of minus-strand RNA levels revealed a defect in minus-strand synthesis (**Fig 3C**). These findings support an important role for the TALS (via its AIL) in mediating replication of RNA1, a property that is akin to the role of TA in facilitating replication of RNA2 [19].

Next, the same three nucleotide substitutions made in R1-172 were introduced into the AIL of the TA in RNA2, generating R2-303 (**Fig 3D**). This modification was made in an RNA2 mutant, R2-MPKO, in which the movement protein (MP) gene was inactivated by a premature stop codon, so that changes made to the TA would not affect corresponding coding of the MP. In contrast to the complete inhibition seen for the TALS modification in R1-172, the equivalent changes in the TA in R2-303 resulted in only a partial defect at both the positive- (~62%) and negative-strand (~72%) levels (**Fig 3E and 3F**). Thus, although both AILs mediate replication of their respective genome segments, the same modification is less debilitating for RNA2.

### Both the AIL and terminal loop of the TA contribute to RNA2 replication

**Fig 4:**
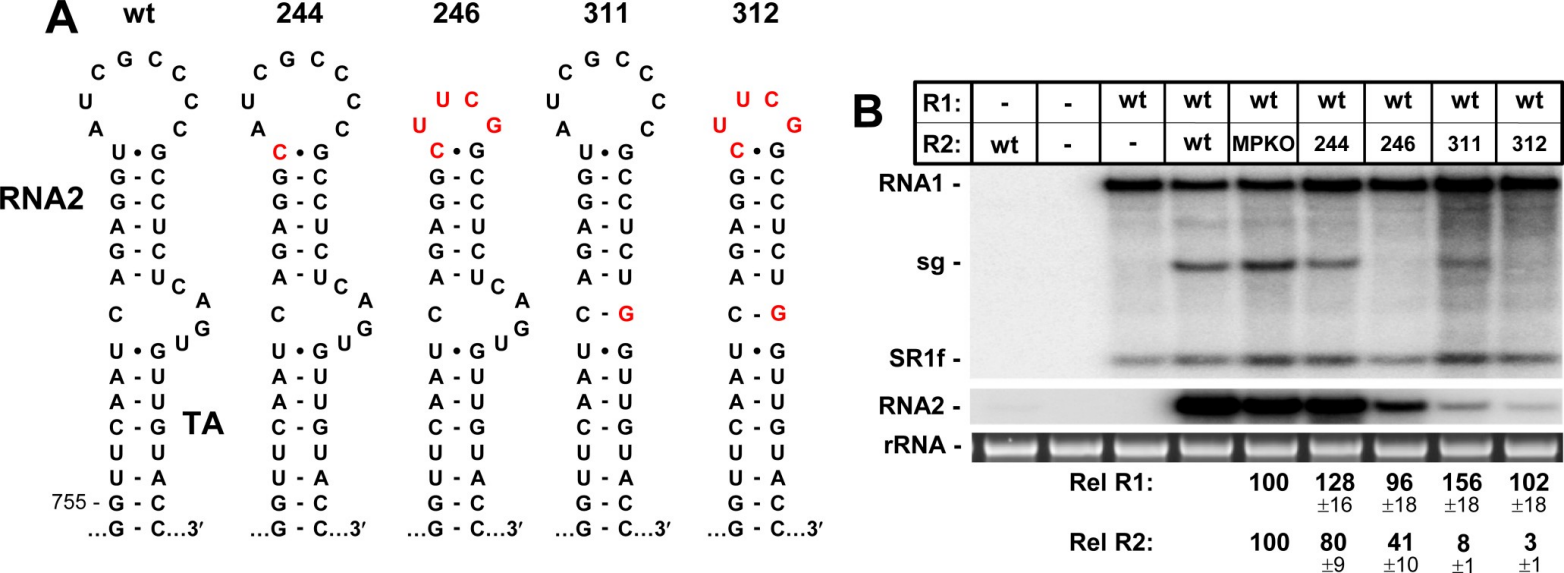
Mutational analysis of the terminal loop and AIL of the TA in RNA2. **(A)** Wt and mutant TAs in RNA2. **(B)** Corresponding northern blot analysis of RCNMV RNA accumulation in plant protoplasts.

The functional resistance of the TA’s AIL to modification raised the question of its importance for RNA2 replication. Similarly, the potential contribution of the terminal loop of the TA to RNA2 levels was also of interest. The RNA2 mutants that were constructed to address these questions, as well as all other RNA2 mutants analyzed in this study, were made in an R2-MPKO background. Accordingly, R2-MPKO served as the “wt” 100% control for quantitative comparisons. In preparation for replacing the terminal loop of the TA with a cUUCGg super-stable tetraloop, the closing base pair in the wt 8-nt-loop was changed from UG to the required CG, creating R2-244 (**Fig 4A**). The single nucleotide change in R2-244 resulted in a modest reduction in RNA2 levels to ~80%, whereas replacement of the wt loop with the tetraloop in R2-246 reduced accumulation to ~41% and, as anticipated, abolished activation of sg mRNA transcription (**Fig 4B**). The observed level of R2-246 accumulation is consistent with another study which showed that substitutions within the TA terminal loop reduce RNA2 levels by ~50% [19]. Eliminating the AIL in R2-311, by replacing its 3ꞌ-half with a guanylate, led to a more drastic decrease in levels to ~8%, however sg mRNA transcription was maintained at a moderate level, indicating that the AIL is not required for *trans*-activation of transcription. Finally, combining the loop and AIL modifications in R2-312 resulted in an additive negative effect (~3%) (**Fig 4A and 4B**). Together, these data indicate that the AIL is the most important subcomponent of the TA for RNA2 replication, with the terminal loop contributing less substantially to the process.

### The TALS terminal loop also facilitates RNA1 accumulation

**Fig 5:**
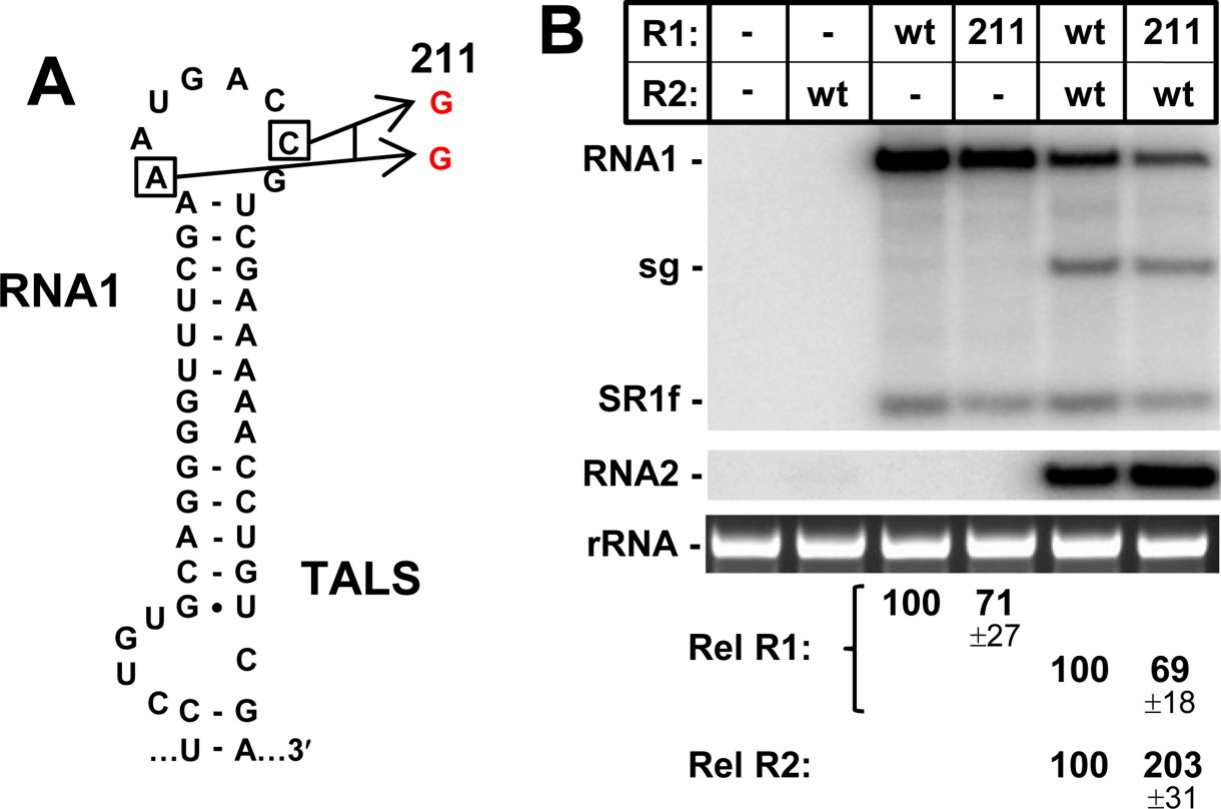
Mutational analysis of the terminal loop of the TALS in RNA1. **(A)** Wt and mutant TALS in RNA1. **(B)** Corresponding northern blot analysis of RCNMV RNA accumulation in plant protoplasts.

The importance of the TA’s terminal loop for its activity suggested that the corresponding terminal loop of the TALS might also be functionally relevant. To test this possibility, two nucleotides, that could be exchanged silently, were substituted with alternative residues, creating R1-211 (**Fig 5A**). A third silent substitution between these residues was not possible, because the corresponding amino acid was a singly-coded methionine. When R1-211 was transfected alone or in cotransfections with wt RNA2, both led to decreased levels of RNA1 to ~70% (**Fig 5B**). Also notable was a twofold increase in RNA2 in the cotransfection, suggesting that the R1-211 defect rendered RNA2 more competitive for replication (**Fig 5B**). Therefore, similar to the TA, both the AIL and terminal loop of the TALS contribute to its activity, but the former substructure appears to be more functionally relevant.

### The AILs of the TALS and TA are functionally interchangeable

**Fig 6:**
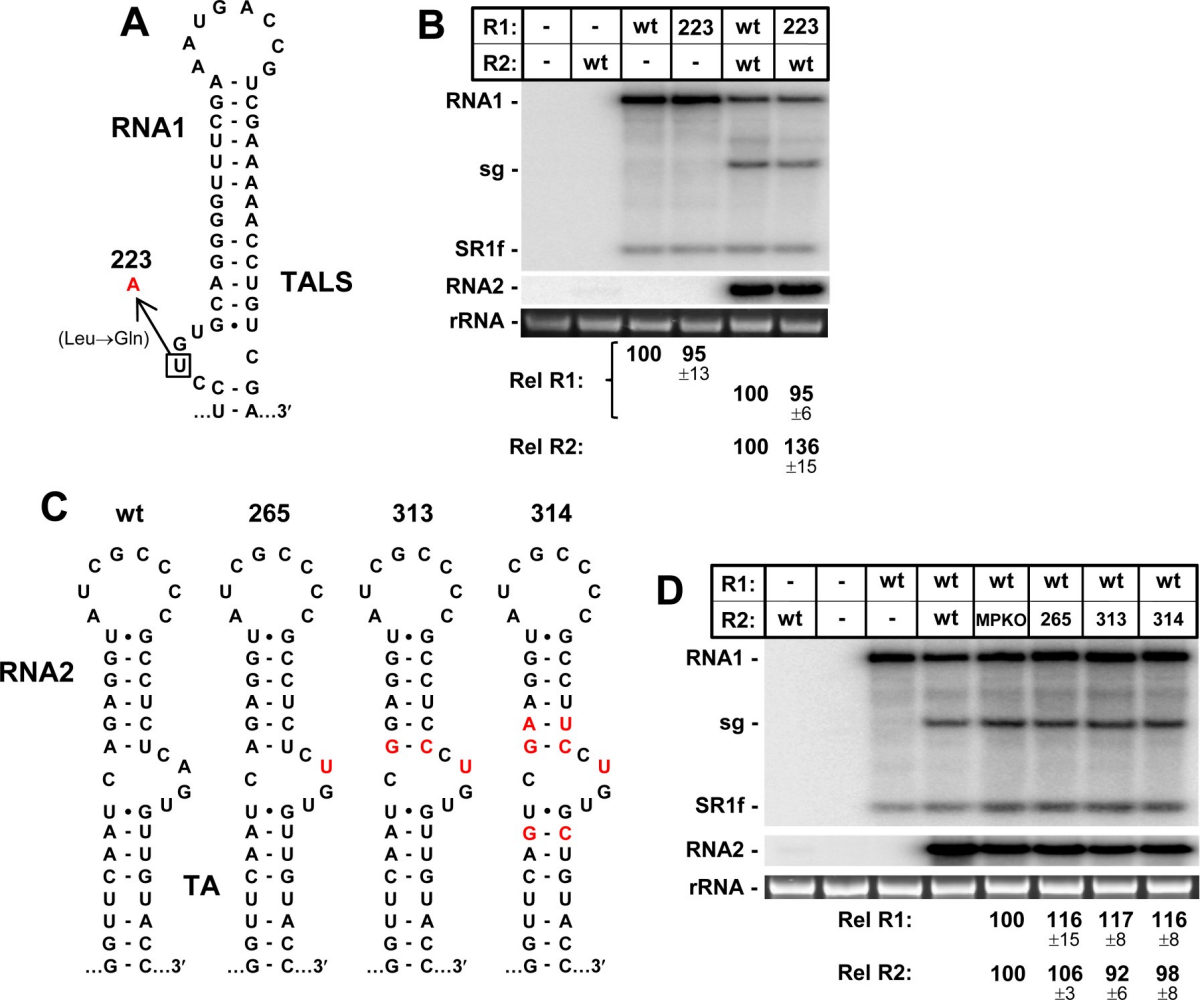
Effect of exchanging the AILs of the TA and TALS. **(A)** Single nucleotide substitution (in red) in the TALS’s AIL in RNA1 that converts it to the TA’s AIL. **(B)** Corresponding northern blot analysis of RCNMV RNA accumulation in plant protoplasts. **(C)** Wt and mutant TAs with substitutions in the AIL and adjacent region in RNA. **(D)** Corresponding northern blot analysis of RCNMV RNA accumulation in plant protoplasts.

Having established a major role in replication for the AILs in the TALS and TA in their respective genome segments, the next unknown to be addressed was the possibility of their functional equivalence. To this end, the AIL in the TALS was converted to the TA’s AIL with a single nucleotide substitution in R1-223, which concurrently resulted in a leucine to glutamine substitution in p88 (**Fig 6A**); however, this amino acid position in the RdRps of tombusvirids is highly variable [23]. R1-223 replicated to near-wt levels in both single and cotransfections with wt RNA2, indicating that the TA’s AIL worked efficiently in the context of the TALS in RNA1 (**Fig 6B**). For RNA2, due to the absence of coding constraints in R2-MPKO, AIL substitutions that also included flanking regions were possible (**Fig 6C**). In R2-265, only the AIL of the TALS was introduced, while in R2-313 and R2-314, the AIL and additional adjacent sequences were added. In all cases, the RNA2 mutants containing TALS-like AILs replicated to near-wt levels (**Fig 6D**). Accordingly, the AILs of the TALS and the TA are functionally exchangeable.

### The TALS and TA are not functionally exchangeable

**Fig 7:**
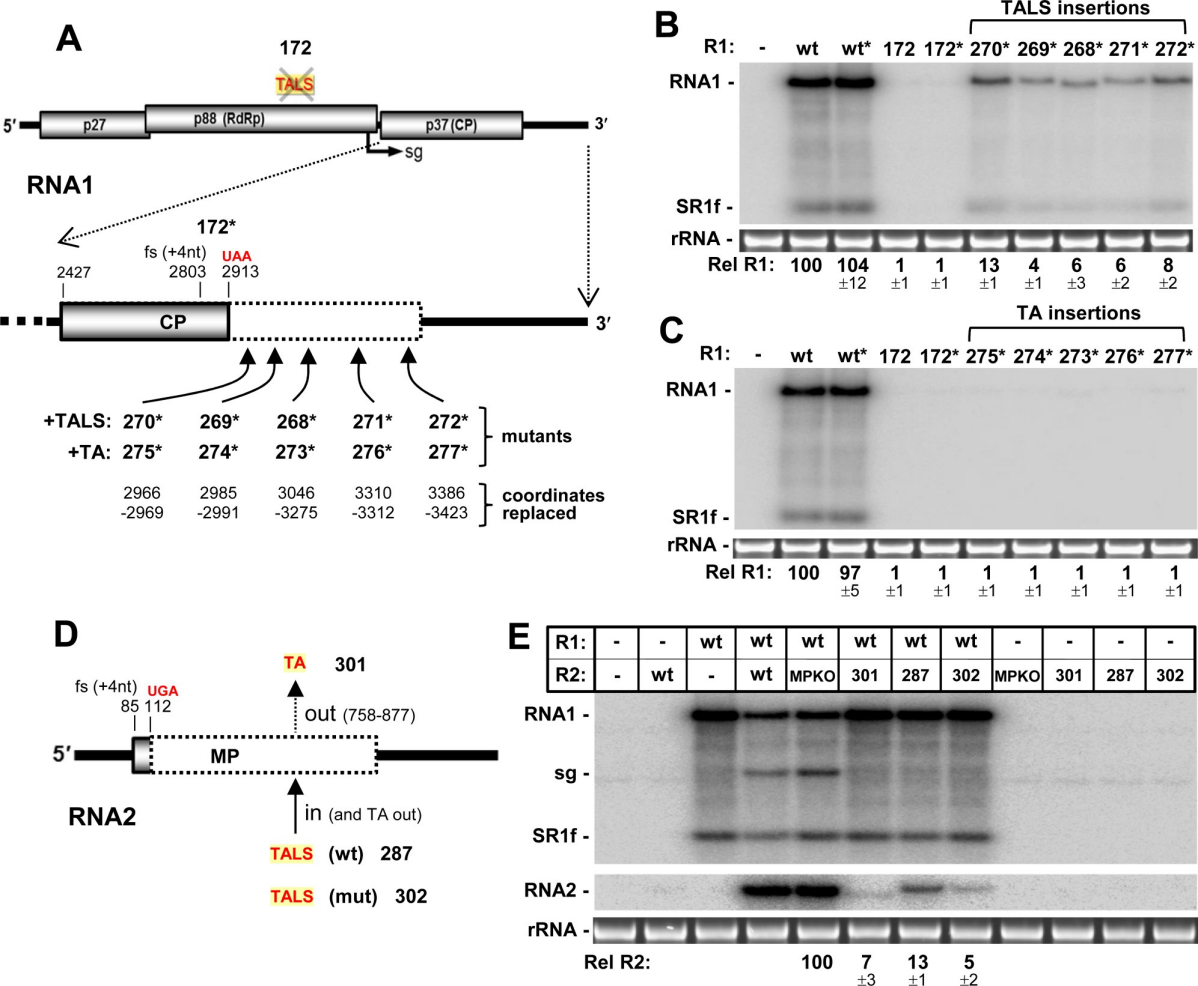
Effect of exchanging the TALS and TA. **(A)** The locations, within the inactivated CP-coding region (dotted box), where TALS or TA sequences were inserted into R1-172* are shown. The names of mutants with TALS insertions or TA insertions are provided in the +TALS and +TA rows, respectively, with the corresponding sequences that were replaced in R1-172* shown directly below. **(B)** Northern blot analysis of RCNMV RNA accumulation in plant protoplasts transfected with R1-172* mutants containing a repositioned TALS. **(C)** Northern blot analysis of RCNMV RNA accumulation in plant protoplasts transfected with R1-172* mutants containing an inserted TA. **(D)** Replacing the TA in RNA2 with a wt or non-functional TALS. **(E)** Northern blot analysis of RCNMV RNA accumulation in plant protoplasts transfected with R2-MPKO mutants containing TALS replacements of the TA.

To test if the entire TA could functionally substitute for the TALS, the R1-172 mutant (**Fig 3A**) that had its TALS disabled was utilized. Additionally, the CP coding ORF in this mutant was inactivated by a frameshift mutation, creating R1-172*, which allowed for segment insertions into this region free from CP coding-related complications (**Fig 7A**). Initially, to test the feasibility of rescuing the TALS-defective mutant R1-172*, the wt TALS was inserted at five different positions in the non-coding CP region that were predicted to maintain proper TALS folding, as assessed by Mfold (**Fig 7A**). Upon transfection, these RNA1 mutants (R1-268* through R1-272*) exhibited very low levels of accumulation, with the maximum recovery being ~13%, for R1-270* (**Fig 7B**). These results indicate that the TALS can only function minimally when transposed into a new RNA1 context. Next, the TA was introduced at the same five positions in R1-172*, thereby generating R1-273* through R1-277* (**Fig 7A**). In all cases, no recovery was observed (**Fig 7C**), suggesting that the TA is not able to functionally substitute for the TALS. However, the low levels of recovery observed with the wt TALS insertions (**Fig 7B**) indicate that this assay may not be sufficiently sensitive to detect possible low level TA-mediated replication of RNA1.

Next the ability of the TALS to rescue a TA-less RNA2 was investigated. The R2-MPKO mutant context was used to create three different RNA2 mutants: (i) R2-301, with its TA deleted, (ii) R2-287, with its TA deleted and replaced by a wt TALS, and (iii) R2-302, with its TA deleted and replaced by a defective TALS (as in R1-172) (**Fig 7D**). Deletion of the TA in R2-301 resulted in very low levels of RNA2 accumulation, while its replacement with the TALS in R2-287 led to a modest twofold increase over that for R2-301 (**Fig 7E**). TA replacement with a defective TALS in R2-302 reduced accumulation down to that of the TA-deleted mutant R2-301, consistent with minimal TALS-mediated rescue for R2-287.

### Improved replication of RNA2 containing a TALS with a stabilized AIL

**Fig 8:**
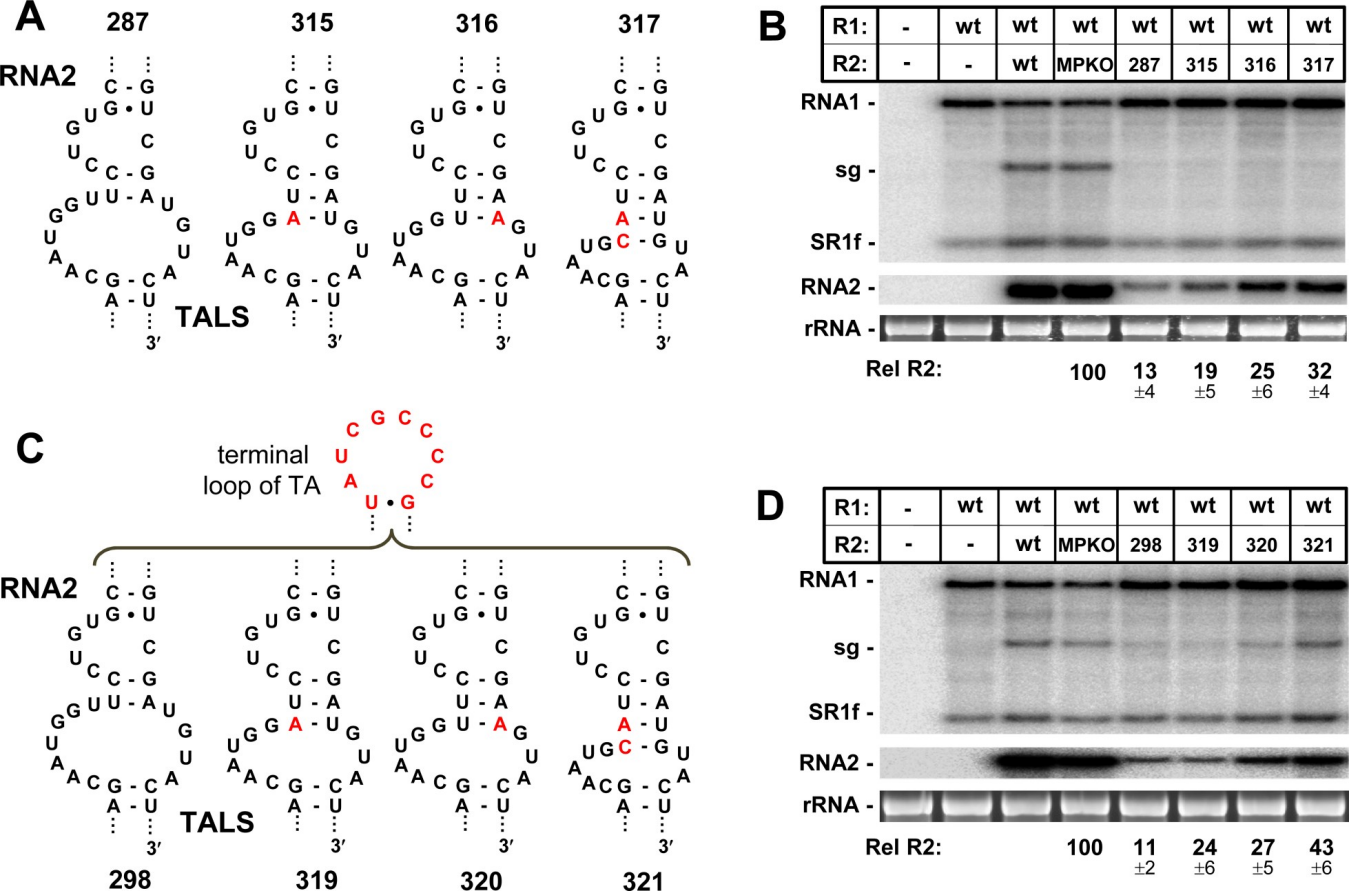
Effect of stabilizing the TALS’s AIL and its context in RNA2. **(A)** Stabilized forms of the TALS’s AIL in R2-MPKO contained nucleotide substitutions that induced additional pairing of the lower stem. **(B)** Corresponding northern blot analysis of RCNMV RNA accumulation in plant protoplasts. **(C)** The mutants in (A) were further modified by replacing their wt TALS terminal loops with that of the TA. **(D)** Corresponding northern blot analysis of RCNMV RNA accumulation in plant protoplasts.

The replacement of the TA’s AIL with the AIL from the TALS allowed for near wt replication of RNA2 (**Fig 6D**), however complete replacement of the TA with the TALS resulted in very low levels of RNA2 accumulation (**Fig 7D**). There are at least two possible explanations for this result. First, the AIL in the TALS is predicted to be less stable than that in the TA, and this could limit the ability of the former to fold properly. To test this idea, the stem below the AIL in the TALS that was inserted into RNA2 was strengthened by one or two canonical base pairs in R2-315, -316 and -317 (**Fig 8A**). These targeted reinforcements resulted in notably improved levels of RNA2 accumulation up to ~32% (**Fig 8B**), supporting improved functionality of the TALS’s AIL in RNA2 when present in a more stable context.

A second possible contributing factor to the minimal activity of the wt TALS in RNA2 could be that its terminal loop is not functionally comparable to that in the TA. To assess this option, the TALS terminal loop was replaced with that of the TA in R2-315, -316, -317, generating R2-319, -320, -321 (**Fig 8C**), this modification modestly improved RNA2 accumulation levels up to ~43%, and led to activation of sg mRNA transcription (**Fig 8D**). The limited enhancement observed suggests that specific spacing or orientation between its AIL and terminal loop may be required, which may not be possible in the TALS’s context.

### Packaging capabilities of the TALS and its AIL

**Fig 9:**
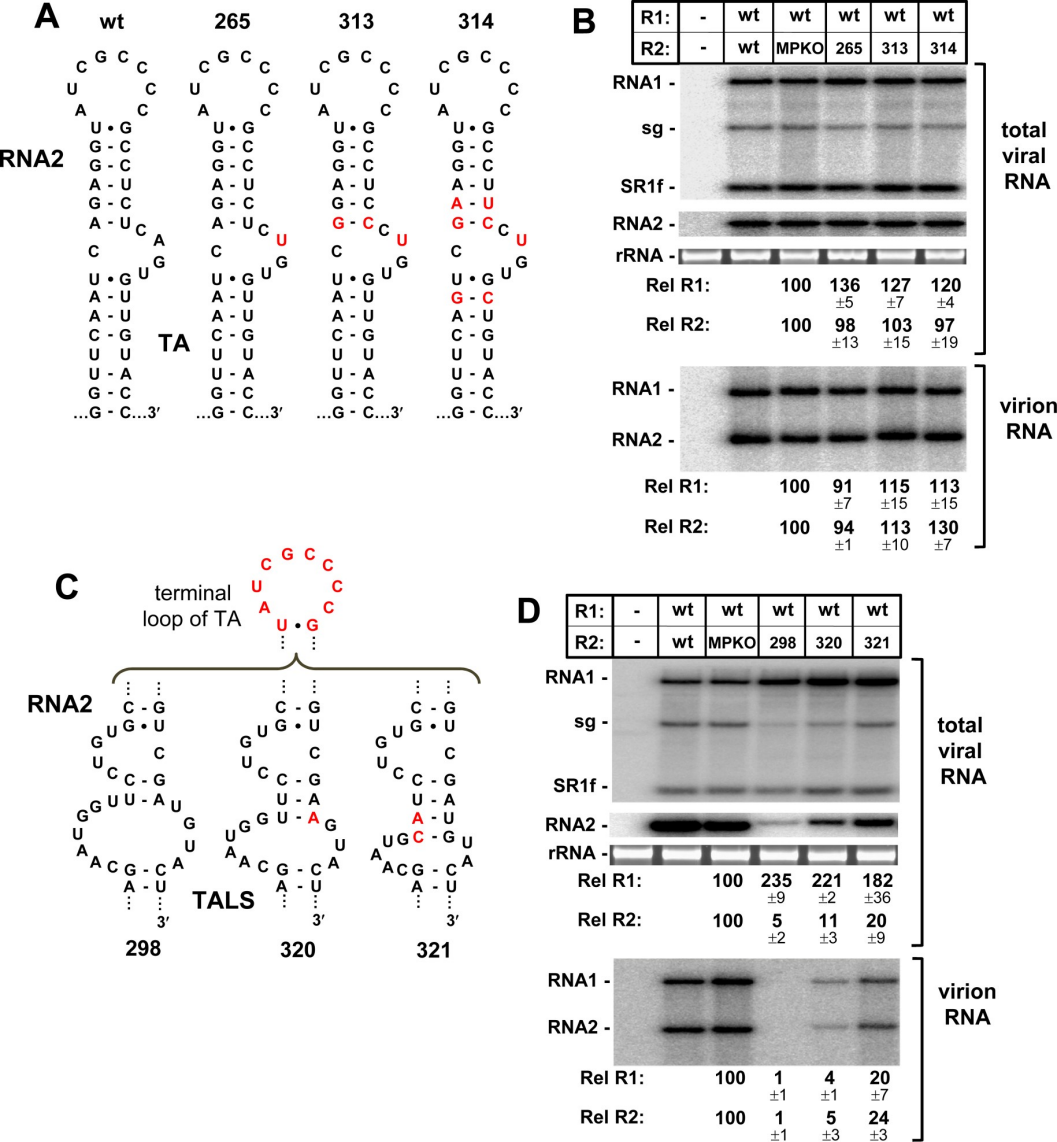
Packaging efficiency mediated by the TALS’s AIL. **(A)** Wt and mutant TAs in R2-MPKO with substitutions in the AIL and adjacent region. **(B)** Corresponding northern blot analyses assessing total viral RNA accumulation and virion RNA levels. Aliquots from the same protoplast infection were used to assess total RNA accumulation and for virus purification. Virion RNA was extracted from particles and separated by agarose gel electrophoresis prior to northern blot analysis. **(C)** TALS mutants in R2-MPKO containing the TA’s terminal loop and stabilized AIL. **(D)** Corresponding northern blot analyses assessing total viral RNA accumulation and virion RNA levels.

The TA has been implicated in nucleating particle assembly and facilitating copackaging [16, 17]. To determine if the AIL of the TALS could functionally substitute for these packaging-related functions, RNA2 mutants R2-265, -313 and -314, with TA’s containing the AIL and surrounding sequences from the TALS, were tested for assembly activity (**Fig 9A**). Each protoplast transfection was assessed for total viral RNA accumulation and corresponding packaging efficiency. For all of the mutants, near wt levels of viral RNAs were detected in transfections along with efficient packaging of both genome segments (**Fig 9B**), indicating that the TALS’s AIL and its context are compatible with virion assembly.

Next, RNA2 mutants with their TA replaced by the TALS were assessed. The TALS in these mutants contained either a wt (R2-298) or stabilized (R2-320 and R2-321) AIL, in addition to the TA’s terminal loop (**Fig 9C**). The latter modification was necessary (i) to allow for a TA-TABS-like interaction to activate sg mRNA transcription, thereby providing coat protein, and (ii) for facilitating copackaging of the two viral genomic segments. Low levels of packaged RNA were observed in all cases (**Fig 9D, lower panel**), with efficiencies roughly correlating with corresponding overall accumulation levels of RNA2 (**Fig 9D, upper panel**). If the RNA2 level in these infections is considered as a limiting factor for packaging (*e*.*g*. 20% for R2-321), then packaging efficiency in the wt+R2-321 cotransfection (*i*.*e*. 20–24%) is comparable to that in the wt+MPKO control cotransfection (**Fig 9D**). Thus, the results further support the AIL of the TALS as being compatible with assembly and copackaging functions.

## Discussion

We have discovered a new RNA element in RCNMV RNA1, which we have termed the TALS. This extended RNA stem loop structure shares both structural and functional features with the TA in RNA2. Here, we compare and contrast these two RNA elements and discuss evolutionary aspects of their relationship.

### The structure and function of the TALS

The TALS is located in the RdRp ORF, within the frameshifted portion of this coding region (**Fig 1A**). This internal position, and the fact that it overlaps with conserved structural motif-D in the palm domain of the RdRp [23], suggests that the TALS arose *de novo* within this coding sequence. The post-frameshift portion of this ORF encounters lower levels of ribosome traffic, thus the TALS would have opportunities to refold between recoding events. This genomic position is also important for TALS function, because moving it to other locations in RNA1 greatly reduced its activity (**Fig 7A**). It is unlikely that the TALS mediates either translation or ribosome frameshifting, since both processes were still operational when it was inactivated (**Fig 3B**). Instead, the data are most consistent with it facilitating replication of RNA1.

Similar functionally-important extended stem loop RNA structures (*i*.*e*., TALS-like) that contain critical internal loops (*i*.*e*., AIL-like) have been identified in the readthrough regions of RdRp ORFs of other tombusvirids (*e*.*g*., tombusviruses, betanecroviruses, aureuviruses, betacarmoviruses) and, like the TALS, these elements are implicated in assisting viral genome replication [24]. The most well-characterized of these RNA structures, RII-SL [25], is present in the genome of Tomato bushy stunt virus and binds specifically to the viral p33/92 replication proteins [26]. A long-range intra-genomic RNA-RNA interaction then repositions the bound replication proteins to the genome’s 3ꞌ-end, where they combine with other host factors to form a functional replicase complex that initiates minus-strand synthesis [27]. The TALS does not function in this capacity, because corresponding RCNMV RNA replication proteins p27/88 do not interact with it (or with the TA) [28]. This implies that the TALS binds to a cellular factor that assists in the replication process. Various host proteins that function in different capacities to promote RCNMV RNA replication have been identified [29]. Whether one of these proteins, or a different host factor, interacts with the TALS, will be investigated in future studies.

### The TALS versus the TA

The similarities between the TALS and TA at the structural level help to explain their functional correspondence, with both promoting replication of their cognate RNAs *in cis*. Both of these RNA structures also appear to mediate minus-strand RNA synthesis, suggesting a possible role in replicase complex formation or some other early step in genome replication. Regarding their genomic contexts, the two elements are located internally in protein coding regions, which suggests *de novo* origins for both.

The terminal loops and AILs are the structural features most pertinent to the functional correspondence of the TA and TALS. For terminal loop modifications, negative effects on RNA segment accumulation were observed in both structures (**Figs 4 and 5**), with the TA being more sensitive to alteration. However, this difference could be related to the change made to the TA terminal loop being more substantial than that to the TALS. Nonetheless, the results suggest notable roles for both terminal loop sequences, which likely relates to their ability to interact with proteins or other viral RNA sequences. Regarding the latter possibility, a search of the RCNMV genome for complementary RNA sequences for the TALS and the TA (other than the TABS) did not yield any compelling candidates, thus, presently, protein partners for both seem more probable.

Exchanging the AILs between the two genome segments maintained near wt levels of replication for RNA1 and RNA2 (**Fig 6**), indicating fundamental mechanistic equivalence. Even so, the wt contexts of the two AILs are unique, as they are oppositely-oriented with respect to their terminal loops, and the AIL in the TALS is predicted to be less stable. The latter difference could, at least partially, explain why the TALS’s AIL was more susceptible to identical modifications. This disparity in local context could also represent a way for the virus to “fine tune” AIL activity and appropriately balance the replication and accumulation levels of its two genome segments. The critical requirement for the AILs implicates them as key ligands for a *trans*-acting protein(s). As mentioned, previous analysis indicated that the TA or TALS do not bind to viral replication proteins p27/88 [28]. This finding is also in agreement with results from the same study showing that RNA2 contains a different RNA element in its 3ꞌUTR, termed the YRE, that is responsible for binding to p27/88 complexes [28]. The structural and functional similarities between the TALS and TA suggest that they likely bind to a common host factor(s) required independently by each genome segment for replication.

The TA-TABS interaction forms an RNA structure that stalls the RdRp, leading to the generation of a minus-strand RNA template that is utilized for sg mRNA synthesis [13]. The TA’s AIL does not appear to be involved in this transcriptional process, as sg mRNA production occurred readily in its absence (**Fig 4**). Interestingly, the wt TA-TABS interaction is predicted to downregulate the TA’s function in RNA2 replication, because base pairing of the TA’s terminal loop (which is important for this activity; **Fig 4**) to the TABS would make it unavailable for alternative interactions. It is also possible that the proximity of the rest of RNA1 to the TA-TABS interaction sterically hinders access of a candidate host factor to the TA’s AIL. Such inhibition of RNA2 replication by the TA-TABS interaction could be important for coordinating opposing processes; *i*.*e*. replication of RNA2 and sg mRNA transcription from RNA1. Indeed, down-regulating the synthesis of RNA2 minus-strands would prevent the RdRp from disrupting the TA-TABS interaction from the opposite direction, thus allowing for unimpeded production of the sg mRNA minus-strand from the RNA1 template.

A key function proposed previously for the TA is its role as a packaging signal [17]. Genome packaging was still observed when the TA’s AIL and its flanking residues were replaced with those of the TALS (**Fig 9A and 9B**). Consequently, if the TA’s AIL does contribute to CP binding and/or nucleation of assembly [17], then the TALS’s AIL also has these capabilities. Additionally, the TALS (with a stabilized AIL) and a TA-derived terminal loop directed packaging when inserted in RNA2 (**Fig 9C and 9D**). This indicates that any AIL-mediated function in virus assembly does not require either precise spatial arrangement relative to the TA-TABS interface or a specific context for the AIL. If CP does bind to the AIL, it would compete with the proposed AIL-binding host protein(s) needed for genome replication. However, the late expression of CP during infections could function to temporally coordinate binding in a sequential manner.

### Exploring the origin and evolution of the TALS, the TA, and their genome segments

Prior to this study, the TA was viewed as a highly unique RNA element in the RCNMV genome. The discovery of a comparable structure in RNA1 prompts a reevaluation of this element and its origins. Here, we present a hypothetical evolutionary scenario for the emergence of RCNMV that is consistent with our current findings and existing information. Virus packaging is included in this scheme by considering the AIL as a determinant of particle assembly [17].

A reasonable starting point is to ask the question: which appeared first, the TA or the TALS? The probable *de novo* genesis of the TALS within the RdRp ORF, its physical and spatial coupling with the RdRp, and the absolute requirement that the RdRp-encoding RNA1 be replicable, all support the TALS arising first. At this point in its existence, RNA1 may have been the sole replicative substrate of its encoded RdRp, and the expression of CP via sg mRNA transcription could have been directed by a *cis*-mediated mode. From a mechanistic perspective, it is reasonable to propose that sg mRNA transcription originally occurred by a simpler *cis*-mediated process and subsequently evolved to a more complex *trans*-based mechanism with its associated benefits. CP produced by a *cis*-mediated mechanism could be recruited to RNA1 for packaging via its TALS (possessing a more stable AIL), which we have shown is compatible with packaging (**Fig 9**). There are current examples of incomplete (*i*.*e*. non-self-sufficient) viral genomes that require coinfection with another virus to complement missing activities, and some are related to tombusvirids; *e*.*g*. umbraviruses and polerovirus-associated RNAs [30, 31]. Accordingly, earlier in its existence, RNA1 could have adopted a similar semi-autonomous viral “lifestyle”, with its movement functions supported by a coinfecting virus.

In this evolutionary scheme, the complementing viral RNA encoding the movement function would represent the precursor of RNA2. During coinfections with RNA1, the sequence corresponding to its MP-coding region would drift, allowing for the *de novo* formation of a precursor-TA (pre-TA) structure that structurally and functionally mimicked the TALS. The genesis of the pre-TA within the MP ORF would ensure their spatial coupling, and the AIL in the pre-TA would confer packaging competence to the precursor-RNA2, including the possibility of copackaging with RNA1. Additionally, the replication-related activity of the pre-TA would make the MP-coding RNA an attractive template for replication-mediated recombination with RNA1. Recombination, at sites flanking the MP ORF, could introduce terminal promoter elements from RNA1, thereby generating an RNA2-like genome segment; consistent with the presence of similar sequences and structures at the termini of current RNA1 and RNA2 segments [2]. Subsequent transition of sg mRNA transcription from *cis*-activation to TA-dependent *trans*-activation via a TA-TABS interaction would enable timely CP production and facilitate copackaging of the genome segments [16]. Lastly, “tuning” of the TALS’s AIL in RNA1 to be less stable would allow it to balance replication levels of the two genome segments and relinquish packaging duties to the TA in RNA2.

The discovery of the TALS and an unanticipated relationship with the TA has provided novel insights into the current status and conceivable origins of their corresponding genome segments. The hypothetical scenario presented above, which is in accordance with available related information, represents but one possible path to contemporary RCNMV. While it is understood that the proposed pathway cannot incorporate or explain all aspects of the present state of RCNMV, the scheme does provide a plausible evolutionary framework to further explore and test possible origins of this segmented RNA plant virus.

## Materials and methods

### Plasmid construction

cDNA clones of wild-type RCNMV RNA1 and RNA2 genome segments (kindly provided by Tim Sit and Steven Lommel) were used to create all RCNMV clones for this study. Standard PCR-based site-directed mutagenesis was used to create RCNMV mutants, using the Q5 High-Fidelity DNA Polymerase kit (NEB). Sequencing was used to confirm that only the desired modifications were present in mutant constructs. Specific changes introduced into the original RCNMV genomes are shown in the accompanying figures.

### Preparation of viral RNAs

Uncapped in vitro transcripts of SmaI-linearized RCNMV constructs were produced individually using the T7-FlashScribe Transcription Kit (CELLSCRIPT) as previously described [32]. RNA transcript concentrations were calculated by spectrophotometry, and transcript quality was verified by agarose gel electrophoresis.

### RNA secondary structure prediction

RNA secondary structures and Pnum values were predicted at 37°C using Mfold version 3.6 using default settings [22, 33].

### In vitro SHAPE RNA structure probing

Selective 2ꞌ-hydroxyl acylation analyzed by primer extension (SHAPE) was completed as described previously [34]. Briefly, full-length in vitro transcribed RNA1 genomic RNA was refolded and then treated with 1-methyl-7-nitroisatoic anhydride. The reaction products were reverse transcribed using Superscript IV Reverse Transcriptase (Invitrogen). Fluorescently labeled primers complementary to RNA1 (nt 2014–2041) were used to evaluate the region of interest. Raw fluorescence intensity data was analyzed using ShapeFinder [35]. The highest ten peak intensities were averaged, and all raw nucleotide reactivities were normalized using this value. The average relative reactivity of each nucleotide from two in vitro SHAPE experiments was mapped onto Mfold-predicted secondary structure.

### Protoplast infection

Cucumber cotyledon protoplasts were prepared and transfected with uncapped *in vitro*-transcribed viral genomic RNA. Specifically, 3x10^5^ protoplasts were transfected with 3μg of RNA1 and/or 1μg of RNA2. The transfections were incubated under constant light for 22 hours at 17°C. Total nucleic acids were extracted as described previously [32].

### Viral RNA analysis

For plus-strand RNA, total nucleic acid extracts from protoplast transfections were separated in non-denaturing 2% agarose gels. Viral RNAs were detected via northern blotting using two ^32^P-radiolabeled probes complementary to the (+)3ꞌend of RNA1 (nt 3704–3724, and 3789–3809) or RNA2 (nt 1222–1242, and 1321–1342). For minus(-) strand detection, total nucleic acid extracts of protoplast transfections were separated in denaturing 1% glyoxal gels as described previously [36]. Minus-sense viral RNAs were detected by northern blotting with ^32^P-UTP labeled riboprobes complementary to the 3ꞌ-ends of negative-sensed RNA1 (nt 3616–3890) or negative-sensed RNA2 (nt 1491–1449). Products of RNA1 and RNA2 were probed separately, as RNA2 and the subgenomic RNA are nearly identical in size. Viral RNA accumulation was monitored using a Typhoon FLA 9500 variable mode imager (GE Healthcare) and quantified by QuantityOne Software (Bio-Rad). All trials were repeated at least three times and averages were calculated with standard errors.

### Packaging assay

Packaging was assessed in protoplast transfections using 12x10^5^ protoplasts transfected with 12μg RNA1 and/or 4μg of RNA2. A tenth of the transfection was used to purify total nucleic isolation and perform northern blotting. The remaining 90% of the transfection was used to isolate virions. Briefly, protoplasts were suspended in 0.2M NaOAc pH 5.2. Sterile glass beads (212–300μm) were added, and protoplasts were homogenized with a Mini-Beadbeater (Biospec Products) using two 15 sec bursts. The homogenate was incubated on ice for 10 min prior to centrifugation at 11000 x g for 10 min. The supernatant was transferred to a new tube, and the precipitate was re-extracted as above. The combined supernatants were kept on ice for 1 hour, and then centrifuged for 10 minutes at 11000 x g. The supernatant was combined with an equal volume of 40%PEG/1M NaCl, and kept on ice overnight. Virions were collected by centrifugation and re-suspended in 10mM NaOAc pH 5.5. Virion RNAs were extracted by PCI, ethanol precipitated, and separated in non-denaturing 1% agarose gels. Viral RNAs were detected via northern blotting using the above described ^32^P-radiolabeled probes complementary to the 3ꞌ-ends of RNA1 and RNA2. Viral RNAs were quantified as described earlier.

## Supporting information

S1 Fig Replication and transcription scheme for RCNMV. RNA1 and RNA2 genome segments are replicated by the viral p27/88 polymerase complex that first synthesizes full-length (-)strand RNAs complementary to the genome segments (grey dotted lines). These intermediate (-)strand RNAs are then used as templates for synthesis of progeny RNA genomes. Sg mRNA transcription occurs when the TA in RNA2 base pairs with the TABS in RNA1 at high concentrations of the two genome segments. The RNA structure formed by the intermolecular TA-TABS interaction causes the viral polymerase to terminate prematurely during (-)strand synthesis of RNA1, resulting in the generation of a sg mRNA-sized (-)strand RNA (green dotted line). This truncated (-)strand is then used as a template for transcription of sg mRNAs.
(PDF)
