# Supplementary figures and images for: A trans-activator-like structure in RCNMV RNA1 evokes the origin of the trans-activator in RNA2
Source: PLoS Pathog. 2020 Jan 6;16(1):e1008271. doi: 10.1371/journal.ppat.1008271 (PMC6964918; doi:10.1371/journal.ppat.1008271)

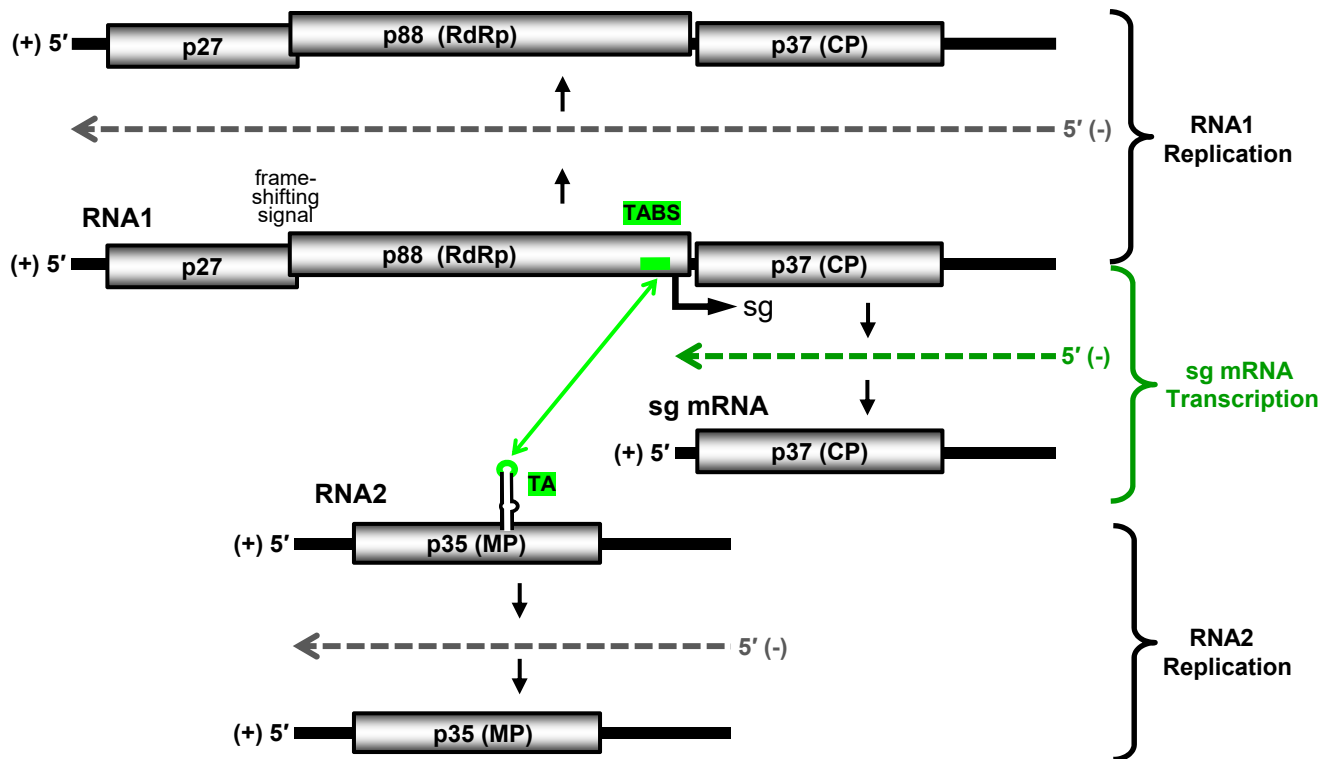

Figure S1. Replication and transcription scheme for RCNMV.

Supplement: S1 Fig — RNA1 and RNA2 genome segments are replicated by the viral p27/88 polymerase complex that first synthesizes full-length (-)strand RNAs complementary to the genome segments (grey dotted lines). These intermediate (-)strand RNAs are then used as templates for synthesis of progeny RNA genomes. Sg mRNA transcription occurs when the TA in RNA2 base pairs with the TABS in RNA1 at high concentrations of the two genome segments. The RNA structure formed by the intermolecular TA-TABS interaction causes the viral polymerase to terminate prematurely during (-)strand synthesis of RNA1, resulting in the generation of a sg mRNA-sized (-)strand RNA (green dotted line). This truncated (-)strand is then used as a template for transcription of sg mRNAs. (PDF) [file ppat.1008271.s001.pdf]
